# An AI-powered navigation framework to achieve an automated acquisition of cardiac ultrasound images

**DOI:** 10.1038/s41598-023-42263-2

**Published:** 2023-09-11

**Authors:** Raska Soemantoro, Attila Kardos, Gilbert Tang, Yifan Zhao

**Affiliations:** 1 School of Aerospace, Transport, and Manufacturing, Cranfield University, Cranfield, MK43 0AL UK; 2 Translational Cardiovascular Research Group, Department of Cardiology, Milton Keynes University Hospital NHS Foundation Trust, Milton Keynes, MK6 5LD UK; 3 Department of Mechanical, Aerospace, and Civil Engineering, The University of Manchester, Manchester, M13 9PL UK

**Keywords:** Echocardiography, Software, Information technology

## Abstract

Echocardiography is an effective tool for diagnosing cardiovascular disease. However, numerous challenges affect its accessibility, including skill requirements, workforce shortage, and sonographer strain. We introduce a navigation framework for the automated acquisition of echocardiography images, consisting of 3 modules: perception, intelligence, and control. The perception module contains an ultrasound probe, a probe actuator, and a locator camera. Information from this module is sent to the intelligence module, which grades the quality of an ultrasound image for different echocardiography views. The window search algorithm in the control module governs the decision-making process in probe movement, finding the best location based on known probe traversal positions and image quality. We conducted a series of simulations using the HeartWorks simulator to assess the proposed framework. This study achieved an accuracy of 99% for the image quality model, 96% for the probe locator model, and 99% for the view classification model, trained on an 80/20 training and testing split. We found that the best search area corresponds with general guidelines: at the anatomical left of the sternum between the 2nd and 5th intercostal space. Additionally, the likelihood of successful acquisition is also driven by how long it stores past coordinates and how much it corrects itself. Results suggest that achieving an automated echocardiography system is feasible using the proposed framework. The long-term vision is of a widely accessible and accurate heart imaging capability within hospitals and community-based settings that enables timely diagnosis of early-stage heart disease.

## Introduction

Cardiac ultrasound, also known as echocardiography, is the first-line diagnostic tool for basic evaluation of cardiovascular disease^1^. Whilst other types of echocardiography exist, the initial assessment of cardiac health mainly relies on transthoracic echocardiography (TTE), which involves manipulating a handheld transducer by a trained sonographer on the surface of the patient’s chest^1^. The challenge for echocardiography is to provide full anatomical and functional imaging of the heart whilst overcoming the different ultrasound properties of various tissues around the heart, such as lung tissues filled with air, bones, blood, muscle, and fat tissues. Although image acquisition is standardised into echocardiographic views, ideal areas of acquisition depend on the position of the heart relative to the ribs and lungs, known as the echo window. Typical cardiac ultrasound views include parasternal long-axis (PLAX), parasternal short axis (PSAX), apical four-chamber (A4Ch), apical two-chamber (A2Ch), and apical three-chamber (A3Ch)^2^. To obtain an echo window image, a trained sonographer or physician applies an ultrasound transducer to the chest, avoiding the ribs and lungs, to guide the beams through deeper structures. The apical and parasternal views can be found approximately between the 2nd and 5th intercostal spaces depending on the patient’s anatomy^2^. The reflected ultrasound signals are captured via the same transducer and reconstructed into an image with the help of a computer. It usually takes at least 10–15 min even for an experienced sonographer to find the right place and angle for the transducer to acquire artefact-free non-foreshortened images appropriate for meaningful clinical use. Moreover, many sonographers have suffered from repetitive shoulder injuries due to regular high-volume scanning^3^ and may be out of work periodically or potentially leave the profession. Therefore, an advanced echocardiography system that can capture data either automatically or be assisted mechanically is highly demanded.

Currently, teleoperated 2D ultrasound systems are commercially available and have been tested for real-world medical uses^4,5^. Such systems, while not strictly requiring an operator in-situ, still require remote guidance from trained sonographers. This has led to the development of automated 2D ultrasound systems, which allow for the acquisition of ultrasound images without or with limited operator intervention. Such systems have been developed for imaging the cervix^6^, foetus^7^, carotid artery^8^, and breast^9^. Note that automated systems differ from assisted systems that guide the user to position the probe correctly, for which a comprehensive review of such systems for cardiac imaging was done in^10^. Except for proof-of-concept studies as in^11^ where a bladder ultrasound was used to image the heart in the case of porcine cardiac arrest, no automated systems have been developed for use in human cardiac image acquisition. This is partly due to echocardiography requiring a specific set of probe handling and positioning techniques that differ in practice and convention from other non-invasive ultrasound acquisitions^9^. Other challenges for automated echocardiography include the use of phased-array transducers in echocardiography and the difficulty of obtaining good images of the heart due to its fast movement compared to other internal organs. The introduction of an automated echocardiography acquisition system would pave the way for fully end-to-end acquisition and analysis systems when used in conjunction with so-called assisted echocardiography methods which detect anomalies and assist diagnosis such as in^12,13^.

With regards to the way the automated methods adjust transducer position, the existing automated 2D ultrasound systems can be categorised into three groups: landmark-based, greedy, and feedback-based. Landmark-based systems use visual input of the body's surface (typically easily distinguishable surface landmarks such as the nipple) to approximate the location to obtain ultrasound images, such as the system in^14^. Greedy systems such as^6,7^ involve acquiring images of a set of regions to generate an overall view of the local anatomy for post-processing. Feedback-based systems employ sensory and/or visual feedback and a set number of rules that govern decision-making in transducer movement^8,15^. Feedback-based systems are theoretically transferable for other types of ultrasound imaging as long as the associated rules are adapted to the specific application.

The rules associated with echocardiography aim to produce an image of a specific cardiac view (e.g., the parasternal and apical views at their intercostal spaces) suitable for diagnosis. The views are typically further classified according to their associated tomographic planes. Several studies have developed systems that classify the view type and image quality, but Pop^16^ stated that standard image classification models like InceptionV3 and VGG16 give a promising baseline accuracy in recognising echocardiography images of various views.

Overall, while automated systems have been developed for medical ultrasound imaging, they have not been developed for cardiac imaging, which has different challenges. Existing automated ultrasound imaging systems use different scanning protocols, such as landmark-based, greedy, and feedback-based approaches, but none are designed explicitly for echocardiography. Feedback-based systems can be adapted for other types of ultrasound imaging by modifying the associated rules. In the case of echocardiography, the goal is to produce images of specific cardiac views that have sufficient quality for diagnosis. The novelty of this work is the introduction of a navigation framework for echocardiography, powered by artificial intelligence, that allows automatically capturing required high-quality images without requiring assistance from radiographers. This research is to pave the way for potential future implementation using a robotic arm.

## Methods

### Conceptual framework

**Figure 1:**
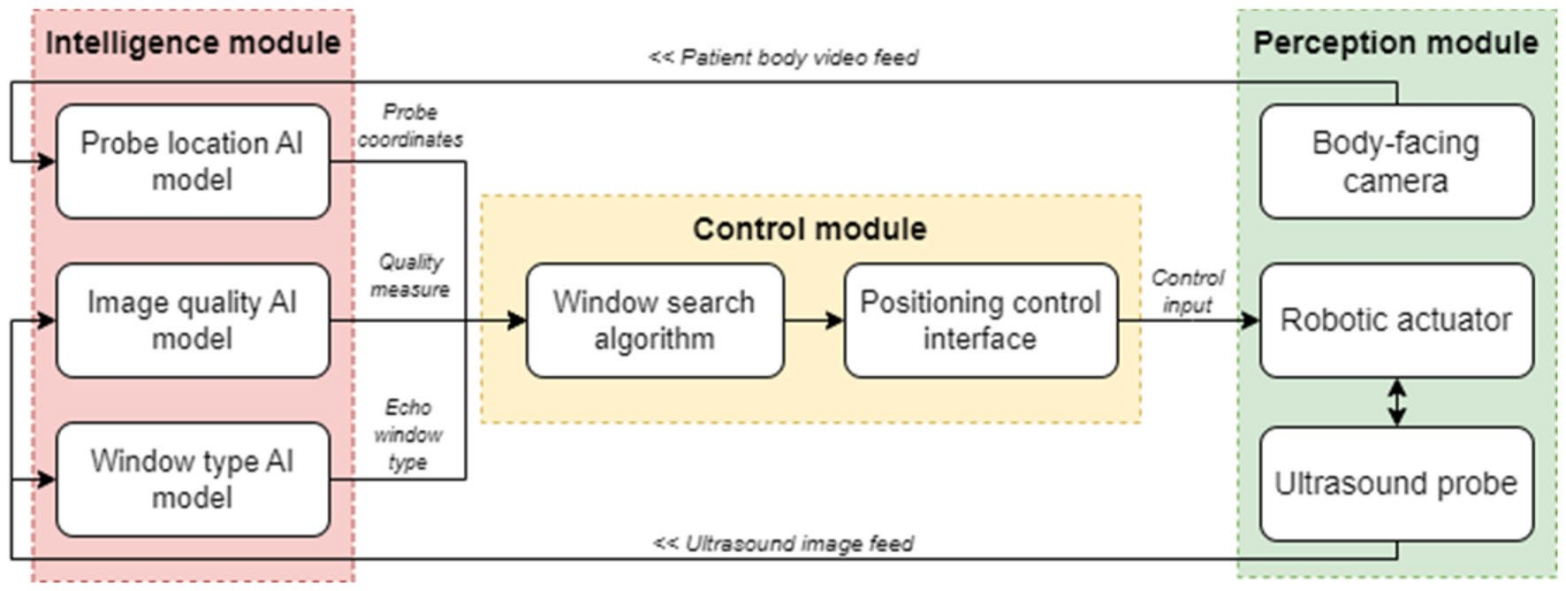
Flowchart of the proposed framework.

The proposed framework consists of the intelligence, control, and perception modules, as visualised in Fig. 1. The intelligence module comprises three distinct artificial neural network (ANN) models: a model to detect the probe location, a model to classify the echo window, and a model to evaluate the quality of live echo images. These models take data from the peripheral devices available in the perception module: the body camera for the probe locator model and the ultrasound probe for the image quality assessment model. The former uses a YOLOv5 object detection model to output the bounding box of the probe in an image from the body camera, while the image quality model employs an InceptionV3 architecture trained to distinguish between ‘good’ and ‘bad’ ultrasound images from manually labelled data.

A main contribution of this research lies in the control module, the key element of which is a novel echo-window search algorithm. This algorithm takes inspiration from the rule-based feedback scanning protocol developed in^8^. It takes echocardiography images and their location descriptors as inputs and outputs the optimal location of the probe in the next step of scanning based on the inputs, with respect to the idealised constraints of the ultrasound positioning system.

Finally, the perception module acts as an interface to the real-world environment, containing a body-facing camera to obtain an image of the current scanning regime, the ultrasound probe as a real-time feed for cardiac imaging, and the robotic arm which manipulates the ultrasound probe’s location. It should be noted that this study provides a conceptual framework for a system that involves a positional controller ideally in the form of a robotic arm. However, for this study, movement is simulated using software, traversing over a set of images with positions that correspond to a physical acquisition on a HeartWorks cardiac ultrasound simulator.

The echo images used in this study were sourced from simulations, publicly available datasets, and those acquired by a cardiologist on a staff member within the cardiology department of Milton Keynes University Hospital who volunteered himself for echocardiography scanning for this study, along with a healthy member from the research team. No patient data was used. The project was approved by Cranfield University Research Ethics System (CURES) with reference number 18154. All confidential information was handled in agreement with the Declaration of Helsinki. All communications were made through National Health Service secure email accounts.

### Implementation

#### System setup

**Figure 2:**
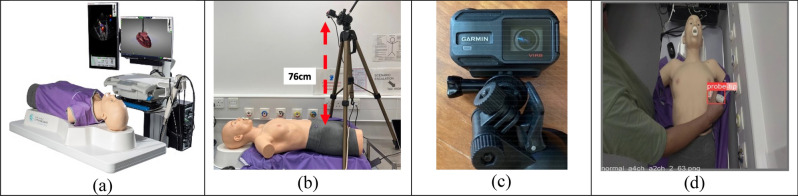
Simulation system setup: (**a**) HeartWorks Simulator; (**b**) body camera setup; (**c**) body camera; (**d**) body camera view.

This study developed a simulation system to analyse the feasibility of the proposed automated echocardiography acquisition framework. Figure 2a shows that the HeartWorks ultrasound simulator, manufactured by MedaPhor Ltd UK^17^, was used to simulate the data capture process of echocardiography. A Garmin VIRB X action camera was placed at a vertical distance of 76 cm to the surface of the patient's torso, parallel to the patient's groin area, facing down, as shown in Fig. 2b. The camera, shown in Fig. 2c, aims to locate the transducer automatically. A typical view of the camera is shown in Fig. 2d.

The location of each captured cardiac ultrasound image is obtained by applying the probe locator model on the videos from the body camera while synchronised to the TTE video. Once done, each image was stored in a file directory according to location (in x–y coordinates) and given a unique image ID. The scanning process is simulated by fetching an image according to the location of its corresponding point. Tests were carried out by varying the step size interval, the search region, and the length of the rolling window. These tests were conducted to obtain a few parameters to evaluate the performance, including success rate, traversal length, simulation time taken and quality of the last image.

#### Image quality assessment model

This model aims to assign a quality metric of a given image obtained in real time through the ultrasound feed. This quality measure is then fed to the control module as input for the window search algorithm. The image quality model employs a convolutional neural network (CNN) to distinguish between ‘good’ (positive) and ‘bad’ (negative) TTE images from a novel echocardiography image quality dataset.

**Figure 3:**
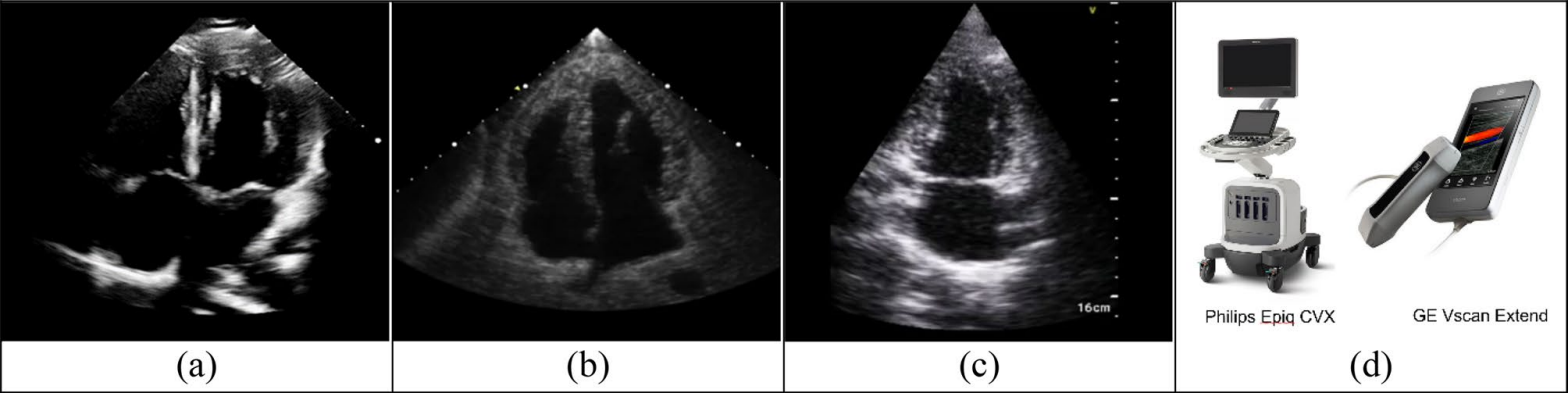
Example ‘good’ images to construct the dataset for image quality evaluation from (**a**) Medical standard device—Philips Epique CVX; (**b**) Heartworks simulator; (**c**) portable device—GE Vscan Extend; (**d**) devices.

To train the model, an image quality dataset containing 5166 images (2852 ‘bad’ images, 2314 ‘good’ images) was used. The first part of the image quality dataset comprises 1137 anonymised images (22% of the whole dataset) collected from Milton Keynes University Hospital using a medical cardiac ultrasound system (Philips Epique CVX). Figure 3a shows an example image with four chambers. The second part of this dataset comprises 3679 synthetic images (71% of the total dataset) sourced from the HeartWorks ultrasound simulator, an example of which is shown in Fig. 3b. The third part of this dataset comprises 346 anonymized ultrasound images (7% of the total dataset) collected by a portable GE Vscan Extend ultrasound device for a healthy participant. Figure 3c shows an example of this in the A2Ch view. The Philips and GE Vscan devices are shown in Fig. 3d. Labelling for this dataset was done manually with reference to views already manually labelled by healthcare professionals.

The CNN architecture implemented in this classification model is the InceptionV3 architecture. This architecture was selected following recommendations presented in^16^, using the InceptionV3 model, which comprises 13 layers and takes an input size of 299 × 299 × 3. Implementation of this model within the proposed system was achieved using the TensorFlow machine learning package, which comes with the InceptionV3 model pre-packaged in its *keras.applications* sub-module.

Before training, the dataset's images were resized into a suitable size (299 × 299 pixels) and then normalised between 0 and 1. This step improved pixel data distribution and convergence time during network training. The model's network weights were initialised on the ImageNet training checkpoint and then trained for 200 epochs on an 80/20 (train/validation) split of the image quality dataset. Table 1 provides the configurations used to train the model. An additional final gate comprising two sigmoid layers was added to the InceptionV3 architecture in^18^ to serve as an output layer, which also serves as a probabilistic representation of the classification process. On inference, the model will output the probability that an image is either a ‘good’ or ‘bad’ quality image. If an image is determined as ‘bad’, the model outputs the image quality by multiplying the probability by − 1. The model outputs the image quality by multiplying the probability by 1 if an image is identified as ‘good’ quality. The closer the quality score is to 1, the better the model predicts the image to be a ‘good’ quality image, and inversely the closer it is to − 1, the lesser the quality of the image. This convention was used to store both label and probability data as a single value. This value is then sent to the window search algorithm within the control module.

**Table 1:** Image quality assessment model training parameters.

Parameters	Value
No. of training epochs (set)	200
No. of training epochs (true)	156
Batch size	32
Optimiser	Stochastic gradient descent
Loss	Sparse categorical cross entropy
Learning rate	0.00001
Momentum	0.9
Early stopping condition	Yes, on no improvement in validation loss during 3 consecutive epochs

#### Echo-window classification model

**Figure 4:**
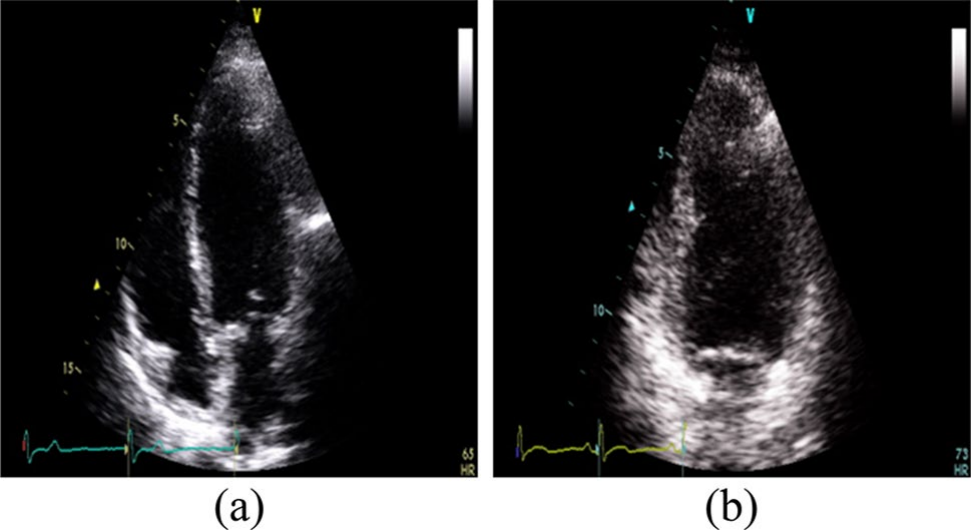
Examples of images for the echo-window classification model. (**a**) Apical 4-chamber. (**b**) Apical 2-chamber.

This classification system differentiates images from two TTE echo windows: the apical 4-chamber (A4Ch) and 2-chamber (A2Ch) views, the principle of which can be extended to other types of echo windows. The CNN used in this model is almost identical to the image quality assessment model but with a different dataset. This dataset comprises 12,980 images from the HMC-QU public echocardiographic videos dataset^19^. Videos are categorised into A4Ch and A2Ch views, which were cut into frames with 6,500 A2Ch images and 6480 A4Ch images. All images in this dataset have a size of 299 × 299 pixels. Sample images are shown in Fig. 4. These views were selected because it is possible to derive a measure of heart function using only these two windows^20^. Furthermore, both views are acquired from the same position; rotating the transducer on the position of the apical 4-chamber view anticlockwise gives the apical 2-chamber view. Note that this research focused on the simplification of the echocardiography image search problem into a 2-dimensional domain, which does not include transducer rotational angle. Once the pre-processing step was complete, the window classification model was trained for 150 epochs using an 80/20 training/validation split of the HMC-QU dataset. The training configurations for this model are given in Table 2. The main difference between this classification model and the image quality assessment model is the output scheme. The output of this scheme is a probability score that indicates how likely a TTE image is to be from an A4Ch view or an A2Ch view.

**Table 2:** Window classification model training parameters.

Parameter	Value
No. of training epochs (set)	150
No. of training epochs (true)	118
Batch size	32
Optimiser	Stochastic gradient descent
Loss	Sparse categorical cross entropy
Learning rate	0.00001
Momentum	0.9
Early stopping condition	Yes, on no improvement in validation loss during 3 consecutive epochs

#### Probe locator model

The dataset for this model comprises 149 images of a simulated TTE examination on the HeartWorks simulation suite. Images were manually labelled using bounding boxes, indicating the ‘probe’ and ‘probe-tip’ using the web-based image labelling software Make Sense^21^. An example is shown in Fig. 2d. All images within this dataset are 1080 × 1080 pixels, each with a *.txt* file describing the bounding boxes shown in the image in the YOLO format: the object class, coordinates, height, and width of the bounding box. The images have been resized into that required in YOLO format and are not in their original aspect ratio.

The model used in this application belongs to the YOLOv5 object detection architecture. This method divides the entire image into a grid system—each cell within the grid is responsible for detecting objects within itself^22^. The specific model architecture used in this implementation is YOLOv5s, which was selected because it offers a good balance between inference time and accuracy. The probe locator model was trained for 200 epochs on an 80/20 training/validation split of the manually labelled probe location dataset. The training parameters used for this implementation are described in Table 3.

**Table 3:** Probe locator model training parameters.

Parameter	Value
No. of training epochs (set)	200
Best training epoch	104
Batch size	1
Optimiser	Stochastic gradient descent
Other parameters were set to default as in^22^	

It should be noted that this probe localisation system is currently in place for simulation purposes only. Once further developments permit the use of the robotic arm for physical testing, the inverse kinematics functions on the robot will be used in conjunction with this vision-based mechanism to obtain the probe location.

#### Echo-window search algorithm

**Figure 5:**
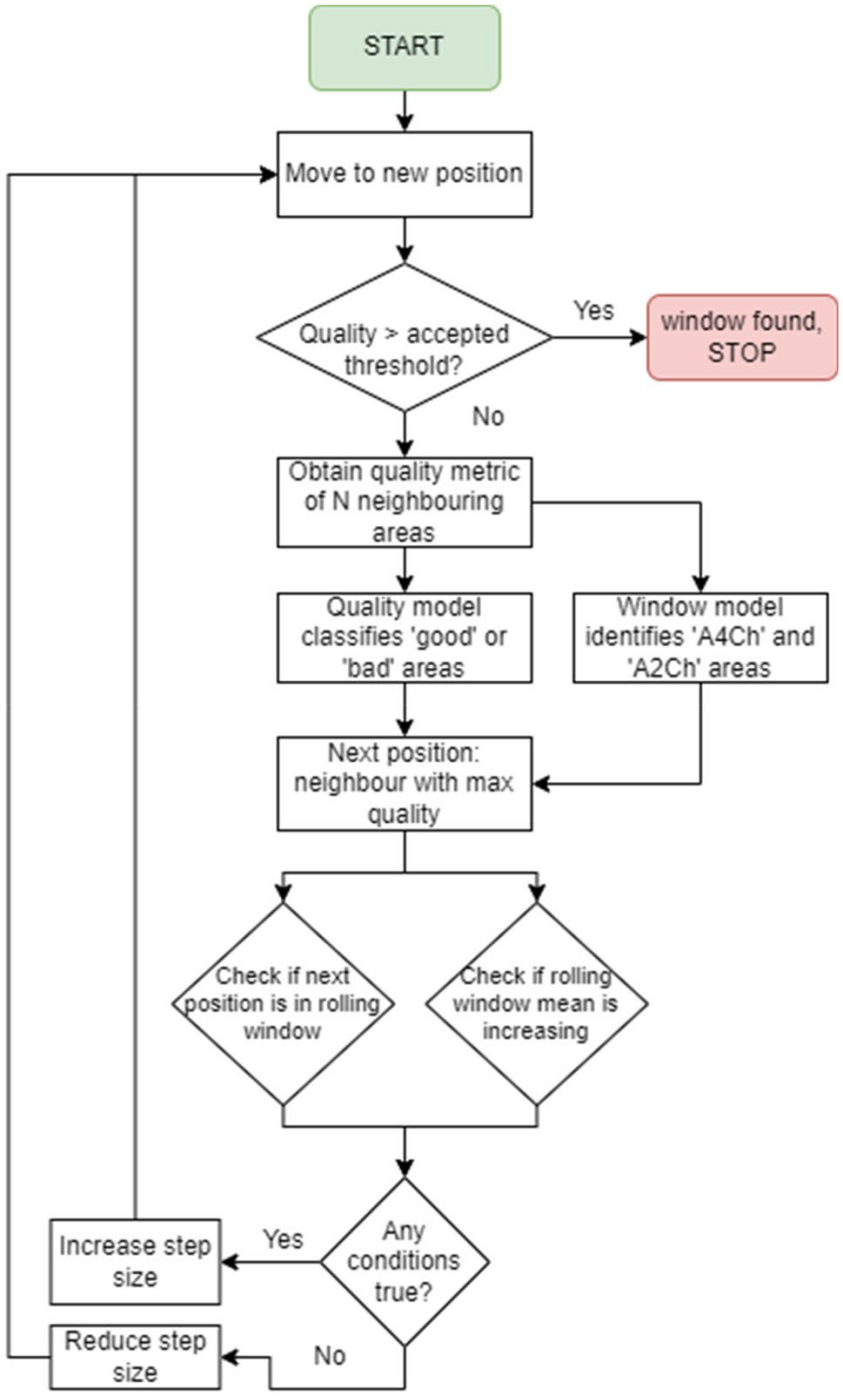
The flowchart of the echo-window search algorithm.

The heart of this proposed system is the window search algorithm, which governs the decision-making process of the robot’s movement. This algorithm finds the best echocardiography window location based on the known traversal positions and corresponding image quality. The proposed algorithm, inspired by the rule-based feedback scanning protocol in^8^, takes echocardiography images and their location descriptors as the input and outputs the optimal scan sequence constrained to the possible movements of the robotic actuator. In the case of a robotic arm, the constraints would differ based on the robot configuration and the degrees of freedom available on the selected system and its working envelope. However, this study has idealised the movements to a two-dimensional space to demonstrate the feasibility of the window search algorithm. The flowchart of this algorithm is shown in Fig. 5.

Firstly, the search region is manually calibrated by the operator, which is done by selecting 4 points on the first image obtained through the body camera feed. By connecting the four points, a polygon can be formed, which denotes the available search area for the window search algorithm, i.e., the window search algorithm's traversal is restricted to only within the boundaries of the 4 points.

Next, a start location is selected—while this start point could theoretically be selected randomly, the ideal start point should follow the general guidelines in performing a TTE examination. This start point is set in the same manner as the search region calibration process using the available user interface. Once this is set, the algorithm commences its iterative search process.

At the beginning of each iteration, the search algorithm checks the image quality at the start point using the image quality assessment system. If the quality meets a preset threshold, the search process will stop. In most cases, the quality of the image at the first step would not meet the threshold. If this is the case, the search algorithm directs the arm to scan the start point's neighbouring positions, i.e., to the north, south, east, and west directions, where north refers to the direction pointing above the transverse plane of the patient and west refers to the direction pointing towards the patient’s anatomical right on the frontal plane. This study refers to a group of these neighbouring positions as the step group. While it is possible to consider a larger step group, this study restricts this to four directions, as mentioned above. As a step in each direction also translates to a change in the pixel coordinates, one can relate the images obtained in each direction with a unique pixel coordinate set. Note that the step size may increase or decrease according to reasons further explained.

Next, the image quality assessment model assesses the quality of all 4 images obtained at each neighbouring position. The algorithm would then store the position corresponding with the best-quality image. This position is chosen as the next starting position in the next iteration. This step also predicts the echo window type to determine whether the image given is of an A4Ch or A2Ch view.

The algorithm employs a set of checks that decide to increase or decrease the step size to improve convergence speed and ensure the search process covers a wider area. This step involves storing the best position and quality score in a so-called rolling window. The length of this window can be varied, as further explained in the simulation testing process.

After the selection of the best position, the algorithm checks whether the position's coordinates are within the rolling window (indicating the arm has traversed this position before) and whether the mean quality score of images within the rolling window has decreased with the addition of the new position (indicating the decision-making process has resulted in obtaining a worse position). If any of the checks are true, it increases the step size in the next iteration, thus avoiding this position in further iterations. If the checks are both false, the step size decreases thus focusing on the area currently traversed. Note that the step size is initialised as 1; however, the amount it decreases or increases is a user-set variable, denoted by the step size interval. The introduction of these checks was necessary for a few reasons: the first check ensures the system is not 'stuck' at a given search area, and the second check ensures that the search algorithm does not waste its time in areas where only sub-optimal TTE images can be obtained.

The next iteration carries over some information obtained in this iteration of the search process, including the step size, rolling window, next start position, and its corresponding quality measure. If the measure meets a set threshold, the traversal ends as mentioned above, but if it does not, the iterative search process continues with new start positions.

## Results and discussion

### Intelligence module

**Figure 6:**
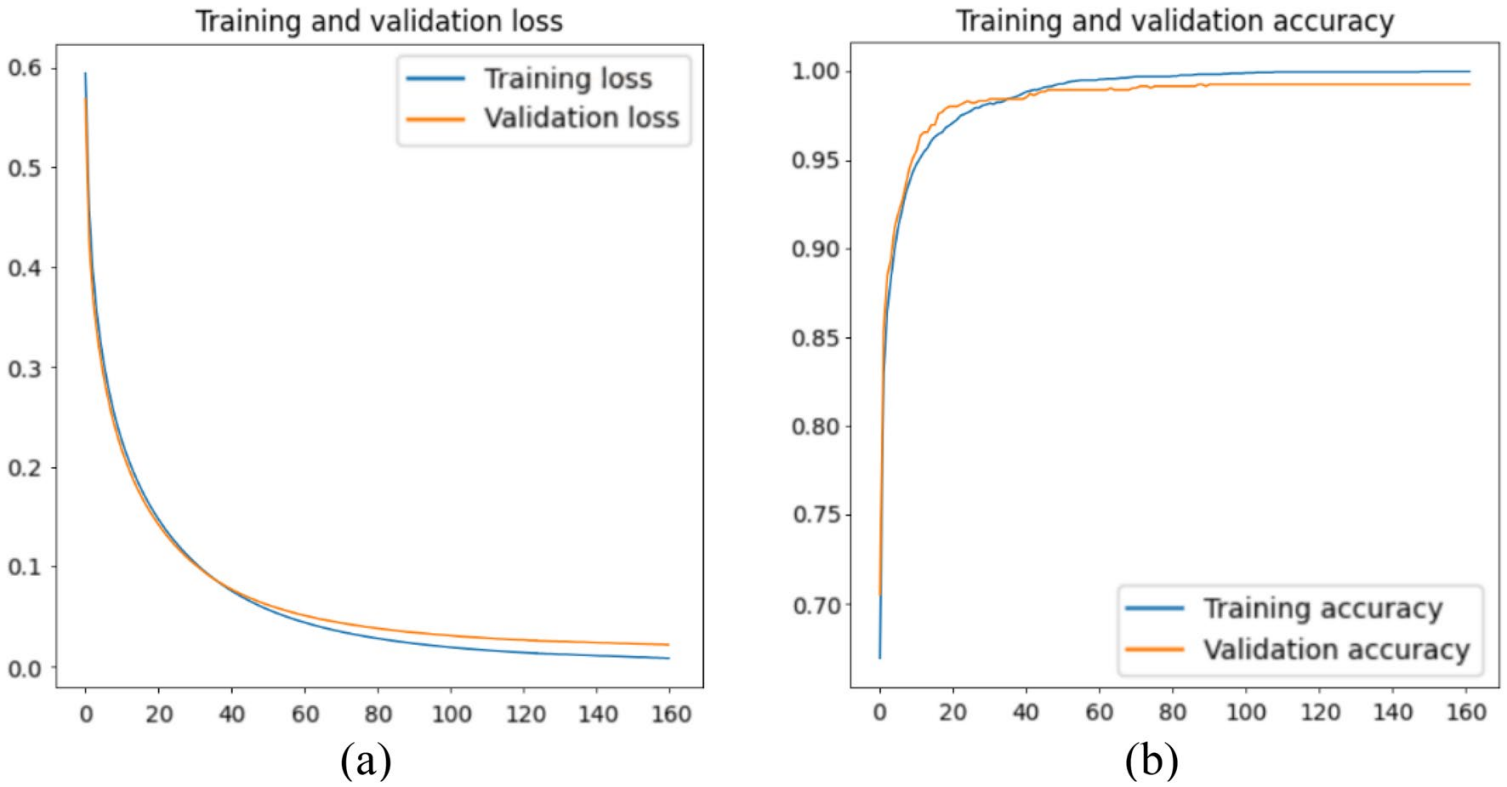
Training and validation (**a**) loss and (**b**) accuracy for the image quality assessment model.

This section presents the results to indicate the performance of the AI models that make up the intelligence module. Figure 6 shows the training and validation loss of the image quality assessment model. The training and validation loss curves both decrease throughout the training process, which indicates a good fit. Also, the accuracy curve increases for both training and validation sets and converges after a certain epoch. Note that the model was set up to stop training prior to a divergence in validation loss to avoid over-fitting.

Table 4 shows the confusion matrix for the validation set. The number of False Negative is slightly higher than that of False Positive, but the overall accuracy is more than 99%. The results from the image classification model are satisfactory for making judgments about image quality. In an ideal scenario, multiple runs with different training parameter configurations should be carried out to find the optimal results. There is potentially some room for optimisation to reduce the risk of over-fitting, however, this study did not focus on fine-tuning the classification process, so additional work is required to achieve the best performance.

**Table 4:** Confusion matrix of the image quality assessment model on the validation set.

	True ‘bad’	True ‘good’
Predicted ‘bad’	0.998	0.014
Predicted ‘good’	0.002	0.986

**Figure 7:**
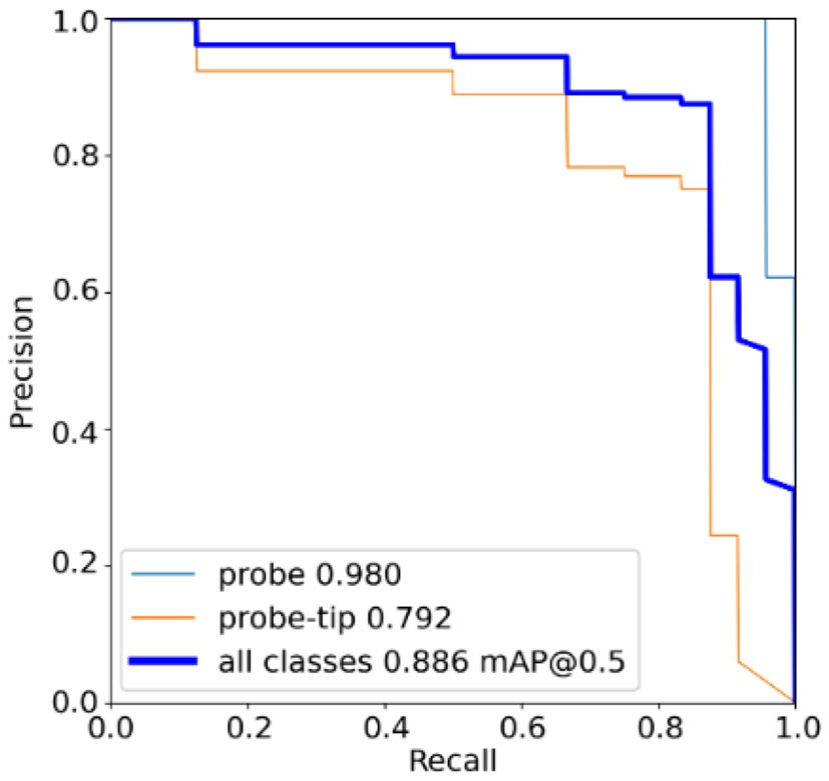
The precision-recall curve of the probe locator model.

Figure 7 shows the precision-recall curve of the probe locator model, and Table 5 shows its corresponding confusion matrix for the validation set. Table 5 and Fig. 7 show that the model can detect probes with high accuracy of 96% but requires optimisation in detecting probe tips. While these training results indicate that there may be room for improvement, it was found that it offered a good balance between accuracy and inference time, which is important when considering the multi-model inference events occurring in each iteration of the proposed system.

**Table 5:** Confusion matrix of the probe locator model.

	True probe	True probe-tip	Background FP
Predicted probe	0.96	0.08	0.29
Predicted probe-tip	0	0.67	0.71
Background FN	0.04	0.25	0

**Figure 8:**
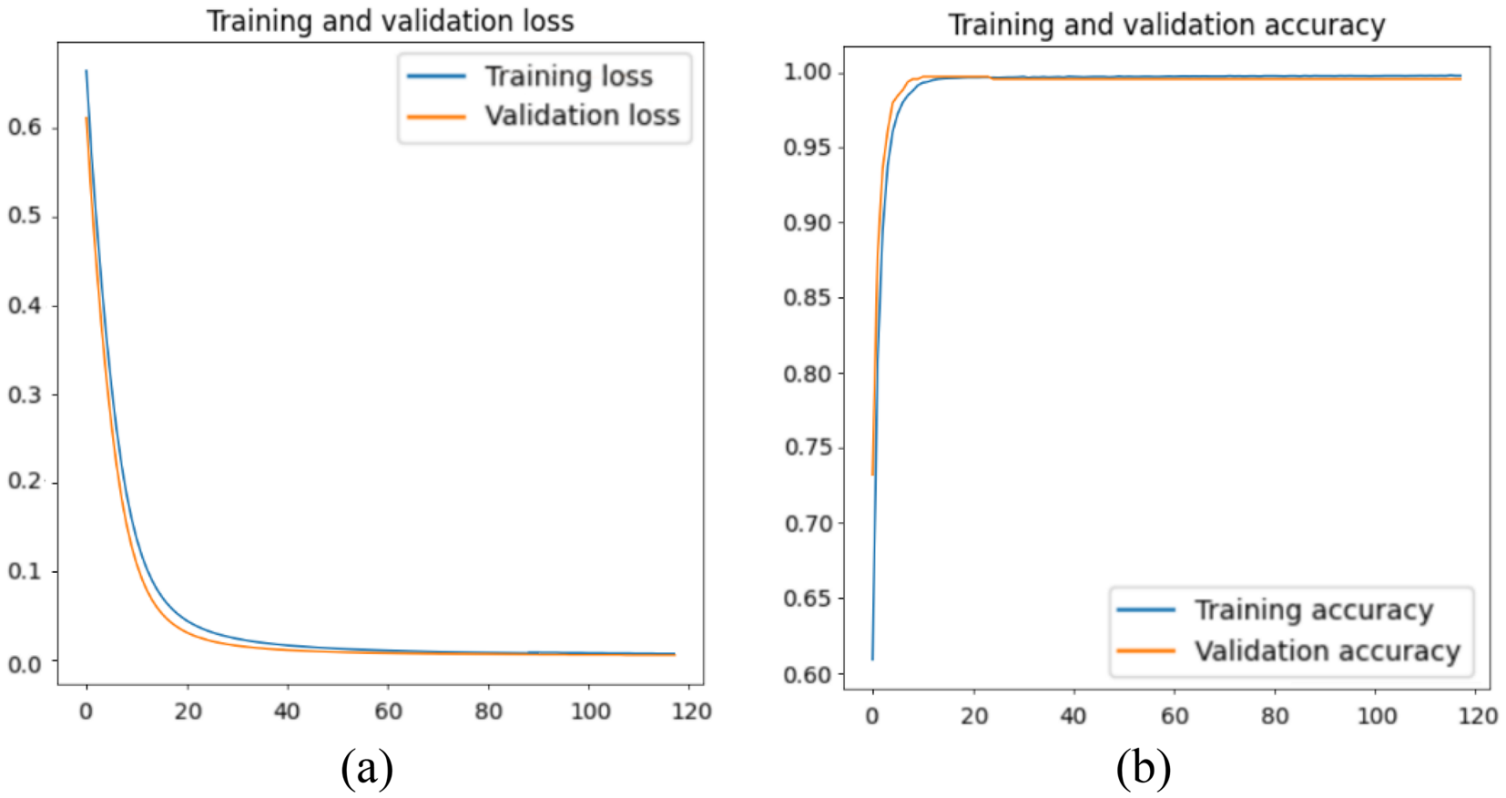
Training and validation (**a**) loss and (**b**) accuracy of the window classification model.

The outcome of the training and validation process of the window classification model can be visualised in Fig. 8 and Table 6. The loss and accuracy curves in Fig. 8 show a similar phenomenon as in Fig. 6, showing a continually decreasing loss and increasing accuracy in window classification. Additionally, the model shows a good final accuracy of over 99% in recognising both apical views, as shown in Table 6. While further improvements can be achieved by tuning the hyperparameters, it is noted that the research scope of this work lies mainly in the proof-of-concept of the search process.

**Table 6:** Confusion matrix of the window classification model on the validation set.

	True A2Ch	True A4Ch
Predicted A2Ch	0.997	0.005
Predicted A4Ch	0.003	0.995

### Control module

#### Search region

**Figure 9:**
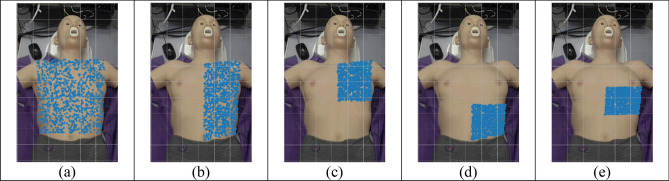
Search regions used in the simulation process: (**a**) Whole torso. (**b**) Left side of torso. (**c**) Left upper torso. (**d**) Left lower torso. (**e**) Left middle torso.

This experiment tests the efficiency of data acquisition by varying the search region size (and thus restricting the starting points) according to the parts of the torso. This is done to showcase the performance of the proposed search algorithm and to understand its behaviour during different calibration scenarios. Images of the different search regions are given in Fig. 9. Other parameters were set as: step size interval: 2; rolling window length: 10; maximum allowed steps: 100; the number of random starting points: 1000. Table 7 gives the results outlining the success rate, the mean quality of the last image, the mean traversal time, and the mean length for each variation of the search region.

**Table 7:** Results of the search domain experiment.

Search region	Success rate	Mean quality of last image	Mean traversal time [s]	Mean traversal length [steps]
Whole torso	0.049	− 0.870	9.59	96.93
Left torso	0.069	− 0.830	9.92	95.68
Upper left	0.123	− 0.736	10.40	91.66
Lower left	0	− 0.958	5.89	100.00
Middle left	0.221	− 0.566	8.70	86.39

The simplest observation to be obtained in Table 7 is that it is impossible to obtain a good echo window by defining a search region in the lower left torso of the patient. This observation is supported by the fact that running 1000 simulations with various start points on the lower left torso area does not yield any successful searches. It is possible to obtain successful window locations using the search algorithm by restricting the search region to either the whole torso, upper left torso, or whole left torso. It is shown that the optimal search region lies in the middle of the left torso. This observation is consistent with the mean quality of the last image, suggesting that setting the search region to the middle-left torso area can yield a higher image quality at the end of the traversal.

Furthermore, the average traversal length and time of the middle-left torso shows that setting the system to search in this region can find the optimal echo window quicker. As Koratala et al.^23^ stated, the location of the apical window is approximately around the 4th or 5th intercostal space, in line with the midclavicular line. Visually this is located slightly towards the anatomical left, below a male patient’s left nipple, or laterally beneath a female patient’s left breast. Considering the anatomy of our ‘patient’ in this case, one can estimate this area to be just around the middle of the torso, towards the patient’s anatomical left. The results in Table 7 show that setting this area as the search domain allows the system to have a higher success rate. As such, the results can be considered reliable, concurring with guidelines for manual acquisition as previously mentioned in^1,2^.

#### Rolling window length and step size interval

**Figure 10:**
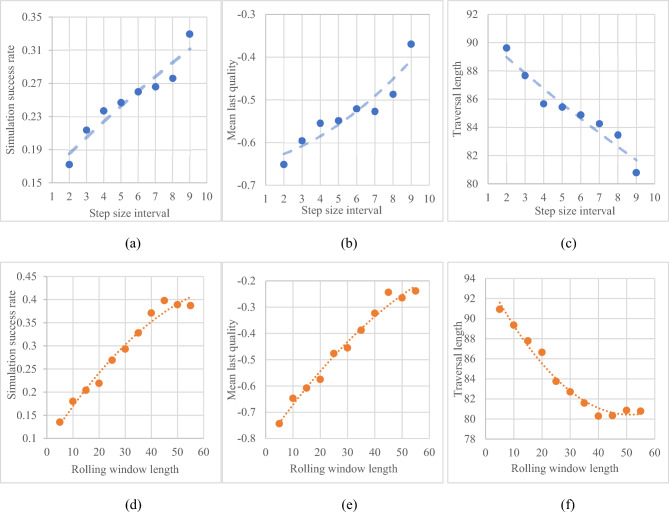
The impact of step size interval and rolling window length on success rate, traversal length and mean last quality, where the scatters denote the measurement and the dash lines plot the fittings.

In Fig. 10, the dots represent the observation’s value for the entire population of search simulations, which are coupled with a second-order polynomial trendline to ease visualisation and help understand the impact of step size and rolling window length on said observed parameters. Figure 10a shows that a higher step size interval would support a higher likelihood of simulation success. This is further shown in Fig. 10b, indicating that traversals that have a higher step size on average end up in a position better suited to obtain a higher quality image. Furthermore, Fig. 10c shows that traversal length is shortened with an increase in step size, which is expected as the system is designed to terminate the traversal if the search succeeds. This observation indicates that the more the search algorithm corrects itself after being penalised for going over a low-quality area, the higher its likelihood of obtaining a good final image. However, the current iteration of this research does not consider simulating how the movement of the arm may affect the traversal time or whether fast repeated movements could be deemed erratic. As the step size interval represents how much the transducer moves during a single adjustment, one could claim that a high step size interval would promote excessive probe movement.

Figure 10d illustrates the relationship between the rolling window length and the success rate of simulations: it is observed that the longer the rolling window, the higher the chance of success. This hypothesis is further supported by Fig. 10e, showing how the quality of images improve as the rolling window length increases. Furthermore, Fig. 10f shows that traversals tend to be shorter when the system records a larger number of past coordinates. These observations are consistent with the design of the rolling window system whose role is to prevent the search from landing on positions it has seen before and move towards better positions to maximize the resulting image quality, and thus reduce the length of time it takes to obtain a good image. The length of the rolling window governs how long it remembers these positions, which can all be deemed sub-optimal as end coordinates are not recorded in the rolling window (i.e., all past steps can be considered mistakes). In essence, it can be considered a short-term memory array that forces the search algorithm to learn from its mistakes—the longer the array, the more it can remember.

#### Start point

**Figure 11:**
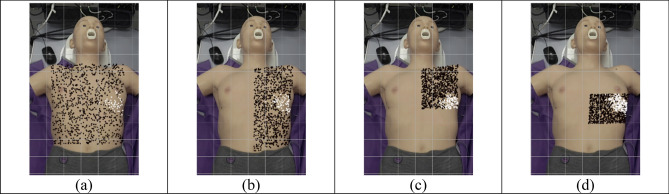
Initial start points for various search domains for (**a**) whole torso, (**b**) left side, (**c**) left upper, (**d**) left middle. Black points represent failed traversals; white represents otherwise.

This section investigates how the start point impacts whether the proposed search algorithm can successfully find a good-quality echocardiography image. Figure 11 shows the impact of the start position across the four different searching domains (The lower left torso is ignored as it ends with no success). Points in each image represent the start position, whereas colour represents whether the traversal ended up successful (black indicates failure, white indicates success). While most simulations were unsuccessful, starting points around the left mid-torso area tend to result in a successful traversal. This area is larger than the footprint of a real cardiac ultrasound transducer, which is designed to fit in a tiny area like the intercostal space. This observation is consistent with existing practical guidelines for where the ultrasound probe should be placed during a real cardiac ultrasound acquisition as in^2^ and^1^.

#### Window type recognition

**Figure 12:**
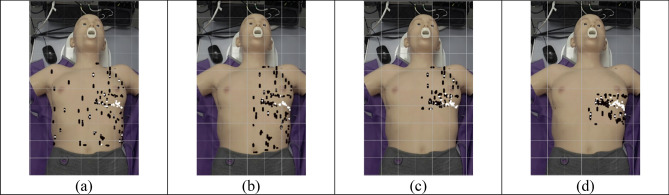
End points for search domain traversals for (**a**) whole torso, (**b**) left side, (**c**) left upper, (**d**) left middle. White points indicate positions for A2Ch and A4Ch imaging, black points otherwise.

The system’s capability to obtain the apical echo window type was investigated. Figure 12 shows the endpoints for various search domains, with the colour representing whether the traversal was completed with an apical image (black indicates false, white indicates true).

It can be observed that while there still exists several failed traversals, the search algorithm outputted A4Ch and A2Ch views in the middle portion of the left torso, occupying a region much smaller than the initial start points. Also, the main distribution of white points does not have a noticeable variation in position on the surface of the patient’s body, the cluster of which forms a slanted thin line on the anatomical left of the torso, akin to the intercostal space, where the desired A4Ch imaging window would be in a real cardiac ultrasound acquisition.

## Conclusion

This research introduced a framework for automated ultrasound image acquisition of the heart. The proposed framework is a feedback-based approach that uses AI algorithms to determine the best location for acquiring images based on feedback from live images. It consists of an intelligence, control and perception module. The main contributions of this paper lie in three AI models in the intelligence module and a window search algorithm in the control module. The framework was implemented and tested using simulations and different data sources. The results suggest the proposed framework can achieve high efficiency and automation if a robot arm is employed. The system could potentially address challenges such as the skill requirements and workforce shortage for echocardiography. It will also reduce the risks of viral transmission and improve the care throughput.

This paper introduces the proof-of-concept of such a solution, where the AI models based on the simulator cannot be used on real-world echocardiography systems directly. For the models to be applied to real-world echocardiography systems, they must be trained on a larger database with considerable subjects and types of echocardiography systems. Other limitations include using a limited simulation environment and the need for consideration of probe depth, imaging settings, and skin contact. It also relies on the calibration of the search region, which requires some training for operators. Future research could address these limitations by constructing a physical platform and performing automated scans on an ultrasound training simulation platform, integrating a force sensor to maintain skin contact, and facilitating an interface to adjust imaging settings. Furthermore, using a robot arm should provide precise step size control and achieve good linearity with the motion.

## Data Availability

Some data that support the findings of this study are available on request from the corresponding author. These data are not publicly available due to containing information that could compromise the privacy of research participants. Some echo image data were sourced from the HMC-QU public echocardiography images dataset which is available at https://www.kaggle.com/datasets/aysendegerli/hmcqu-dataset.
